# Odorant Receptor PxylOR11 Mediates Repellency of *Plutella xylostella* to Aromatic Volatiles

**DOI:** 10.3389/fphys.2022.938555

**Published:** 2022-07-13

**Authors:** Yipeng Liu, Sai Zhang, Yang Liu, Guirong Wang

**Affiliations:** ^1^ Shenzhen Branch, Guangdong Laboratory for Lingnan Modern Agriculture, Genome Analysis Laboratory of the Ministry of Agriculture, Agricultural Genomics Institute at Shenzhen, Chinese Academy of Agricultural Sciences, Shenzhen, China; ^2^ State Key Laboratory for Biology of Plant Diseases and Insect Pests, Institute of Plant Protection, Chinese Academy of Agricultural Sciences, Beijing, China

**Keywords:** *Plutella xylostella*, odorant receptor, two-electrode voltage clamp recordings, behavioral response, repellent

## Abstract

Insects can use plant volatiles to guide certain behaviors, such as courtship, mating, host positioning, and habitat selection. *Plutella xylostella* is a global agricultural pest and has always been closely studied, but relatively few studies assess the molecular mechanism of *P. xylostella* exposed to plant volatiles. In this study, we analyzed the role of the odorant receptor PxylOR11 when *P. xylostella* is exposed to plant volatiles. Our analysis of tissue expression demonstrated that PxylOR11 is expressed in the antennae and that expression levels in female moths were significantly higher than in male moths. Functional analyses using the *Xenopus* oocyte expression system demonstrated that PxylOR11 was tuned to three aromatic compounds: benzyl alcohol, salicylaldehyde, and phenylacetaldehyde. Electroantennogram analyses revealed that these three aromatic compounds can induce electrophysiological responses in the antennae of *P. xylostella*, and that the electroantennograms response value of female moths was significantly higher than that of male moths. Dual-choice bioassays demonstrated that the three aromatic compounds have a repellent effect on female *P. xylostella*. These results suggest that PxylOR11 has a role in mediating the repellent effect of aromatic volatiles on *P. xylostella* and can be used as a potential target to design novel olfactory regulators controlling *P. xylostella*.

## 1 Introduction

Plant volatiles can guide a series of physiological or behavioral responses of insects, including host positioning, oviposition, defense, and habitat selection, and play a decisive role in chemical communication between plants and insects (Payne and Kennedy, 1987; Larsson et al., 2004). The research on the effects of plant volatiles on the behavior of intraspecific and interspecific insects has also attracted more and more attention, which is of great significance for understanding the relationship between plants and insects.

The volatiles released by plants under normal conditions are usually complex mixtures composed of some low molecular weight organic chemicals, such as hydrocarbons, alcohols, ketones, aldehydes and terpenes. Each plant has its own volatiles, and each constitutes the chemical map of the plant in accurate proportion. These chemical maps play an important role in insect host selection (Pettersson et al., 1998). For example, cowpea aphid (*Aphis craccivora*) have the ability to distinguish between cowpea and other host plants. Among these volatiles, they are mainly divided into two categories: One is specific compounds, such as allyl isothiocyanate released by cruciferous plants; the other is general odors, which are common to most plants, usually referring to straight chain alcohols, aldehydes and ester compounds containing six carbon atoms (Lecomte et al., 1998). In addition, plants will produce and release volatiles after being fed by herbivorous insects, which are called herbivore-induced plant volatiles (HIPVs), mainly including terpenoids, green leaf odor, nitrogen-containing compounds, sulfur-containing compounds. These compounds play a very important role in the indirect defense of plants (repelling or attracting natural enemies) (Unsicker et al., 2009; Yang et al., 2016). At present, there have been more and more studies on the relationship between plant volatiles and insect behavior, and many achievements have been applied to pest control, such as plant attractants, repellents, antifeedants, physiological and biochemical inhibitors and disruptors, but the research on the chemical communication mechanism between insect and plant is not deep enough.

The detection of plant volatiles mainly occurs through the olfactory neurons in different types of sensilla on the antennae surface, where odorant-binding proteins (OBPs) are proposed to carry volatiles from the outside to the membrane of olfactory sensory neurons (OSNs), odorant receptors (ORs) are expressed on the dendritic membrane of OSNs for reception of volatiles (Suh et al., 2014). ORs form heteromultimer with a universal OR co-receptor (Orco) to transform volatile chemical signals into electrical signals. These electrical signals are then transmitted to the central nervous system by the antennal nerves and further signal integration produces the appropriate behavioral response (Leal, 2013). Therefore, studying ORs provides important insight into the mechanism regulating the signal recognition of insect chemicals (de Bruyne and Baker, 2008).

The first ORs of insects were found in *Drosophila melanogaster* in 1999 (Clyne et al., 1999; Gao and Chess, 1999). The continuous development of bioinformatics and sequencing technologies has identified an increasing number of insects ORs, especially in Lepidoptera. These include *Helicoverpa armigera*, *Mythimna separata*, and *Ostrinia furnacalis* (Liu et al., 2012; Yang et al., 2015; Du et al., 2018). However, the systematic studies on the function of Lepidoptera ORs are only reported in *Spodoptera littoralis* and *H. armigera* (de Fouchier et al., 2017; Guo et al., 2020). For other Lepidoptera insects, the functional research of ORs mainly focuses on sex pheromone receptors (PRs), and the research is very in-depth (Liu et al., 2013; Liu et al., 2018; Guo et al., 2022). In contrast to PRs, there are relatively few functional studies on general ORs. Large numbers of general ORs have important functions (Liu et al., 2014; Zhang et al., 2016; Zhang et al., 2017). For instance, HassOR67 plays a key role in the recognition of nonanal, an ovipositional cue for *H. assulta* (Wang C. et al., 2020). In *Eupeodes corollae*, general EcorOR25 is tuned to floral scent volatiles (eugenol and methyl eugenol), which is an attractant for adult male and female of *E. corollae* (Li et al., 2020). Similarly, HarmOR42 is the key receptor for *H. armigera* to recognize phenylacetaldehyde, which is a floral scent component common in most angiosperms (Guo et al., 2020). Therefore, research on the function of general ORs will uncover the mechanism regulating insect locating location of host plants and oviposition sites and contribute to the development of new behavioral regulators for controlling pests.

The diamondback moth, *P. xylostella* (Lepidoptera: Plutellidae), is a global pest that attacks brassica (Brassicales: Brassicaceae) crops. The annual global costs due to economic losses inflicted by *P. xylostella* and associated control costs can reach 4–5 billion US dollars (Zalucki et al., 2012; You et al., 2020). *P. xylostella* has a short generation cycle and high fecundity, making it highly resistant to insecticides (Furlong et al., 2013). This makes it difficult to control. Therefore, identifying new ways of controlling *P. xylostella* is important for the vegetable production industry. Many researchers have focused on the PRs of *P. xylostella*, but studying of general ORs provide a better understanding of the host recognition mechanism of *P. xylostella* and provide a new method of controlling it (Sun et al., 2013; Liu et al., 2018; Liu et al., 2020). In this study, we cloned a general OR-PxylOR11 from the antenna of *P. xylostella*. Quantitative Realtime-PCR (qRT-PCR) was used to demonstrate that PxylOR11 was highly expressed in the antennae, and the expression level of the female antennae was significantly higher than that of the male antennae. By combining *In vitro* expression of *Xenopus* oocytes and the two-electrode voltage clamp technique, we found that PxylOR11/Orco responded to three aromatic compounds (benzyl alcohol, salicylaldehyde, and phenylacetaldehyde). Behavioral experiments demonstrated that benzyl alcohol, salicylaldehyde and phenylacetaldehyde have repellent effects on female moths. These three compounds with repellent effect against *P. xylostella* were screened using a reverse chemical ecology approach, which can provide a new and integrated method of controlling *P. xylostella*.

## 2 Materials and Methods

### 2.1 Insects Rearing and Tissues Collections

*P. xylostella* larvae were collected in the suburban of Beijing in 2001 and reared on fresh Chinese cabbage leaves and 70 ± 5% relative humidity at 26 ± 1°C on a 16:8 h (light/dark) photoperiod cycle at the Institute of Plant Protection, Chinese Academy of Agriculture Sciences, China. Adults were fed 10% honey solution, and the adult tissues (genitalia, leg, abdomen, thorax, head and antennae) were collected from 2- to 3-day-old virgin 100 male and 100 female moths in liquid nitrogen and stored at −80°C until use.

### 2.2 RNA Isolation and cDNA Synthesis

Total RNA was extracted from the above tissues using Trizol Reagent (Invitrogen, Carlsbad, CA, United States). The NanoDrop ND-2000 Spectrophotometer (NanoDrop Technologies, Inc., Wilmington, United States) were used to verify the purity and concentration of the RNA, and gel electrophoresis was used to verify the integrity of RNA. Total RNA was treated with DNaseI (TransGenBiotech, China) to remove traces of genomic DNA. Total RNA (1 μg) of the different tissues were used for first single-strand cDNA synthesis used RevertAid First Strand cDNA Synthesis Kit (Thermo Fisher Scientific, United States). The resultant cDNA was used as the templates for gene clone and Real-time quantitative PCR (qPCR). The qPCR experiments were repeated three times using three independently isolated RNA samples.

### 2.3 Gene Cloning and Sequence Analysis

The sequence of odorant receptor (PxylOR11) was identified through transcriptomic results (Yang et al., 2017). Using the male and female antennae cDNA of *P. xylostella* as a template, the open-reading frames (ORFs) of the PxylOR11 was cloned using specific primers (Supplementary Table S1). The specific primers were designed by Primer Premier 5.0 (PREMIER Biosoft International, CA). The PCR reaction was performed in 25 μL including 2 × PrimeSTAR Mix (12.5 μL, Takara, Dalian, China), upstream primers (1 μL, 10 μm), downstream primers (1 μL, 10 μm), cDNA template (1 μL) and ddH_2_O (9.5 μL). PCR reactions were performed under the following conditions: 98 °C for 3 min; 35 cycles of 98 C for 10 s, 56 C for 10 s, 72 C for 90 s; 72 C for 10 min. PCR amplification products were analyzed on a 1.0% agarose gel and ligated with the pEASY-Blunt vector (TransGen Biotech, Beijing, China), and sequenced by Sangon Biotech, Shanghai, China. TOPCONS (http://www.topcons.net) was used for the prediction of transmembrane domains (TMDs). PxylOR11 amino acid sequences and other Lepidoptera species were used to construct a phylogenetic tree (Krieger et al., 2004; Tanaka et al., 2009; Liu et al., 2012). Phylogenetic trees were constructed using RAxML version 8 with the Jones-Taylor-Thornton (JTT) amino acid substitution model (Informer Technologies, Inc.) (Tamura et al., 2013). Node was assessed using a bootstrap method based on 1,000 replicates (Cao et al., 2014).

### 2.4 Real-Time Quantitative PCR

Antennae (A), legs (L), genitals (G), thoraxes (T), abdomens (Ab), and heads (without antennae) of males and females were excised from 2- to 3-day-old *P. xylostella* virgin adults. RNA isolation and cDNA synthesized were performed as mentioned above. qPCR was employed to analyze the expression of PxylOR11 in the different tissues with ABI Prism 7500 Detection System (Applied Biosystems, United States). The 20 μL reactions in including 0.5 μL upstream primers (10 μm), 0.5 μL downstream primers (10 μm), 1 μL cDNA templates, 10 μL of 2 × Go Taq qRT-PCR Master Mix (Promega, United States) and 8 μL H_2_O. The qPCR reaction conditions were set as follows: 95 C for 3 min, 40 cycles of 95 C for 10 s and 58 C for 30 s. Combined with our previous research results (Sun et al., 2013; Liu et al., 2018), the PxylActin (GenBank AB282645) was used to verify the integrity of the above cDNA templates. Three RNA templates from different generations of *P. xylostella* were used three biological replicates. The PxylOR11 relative expression levels were calculated using the comparative 2^-△△CT^ method (Livak and Schmittgen, 2002). The sequences of primers are available in Supplementary Table S1.

### 2.5 Plant Volatile Compounds

The 55 plant volatiles used in the experiment were purchased from Sigma-Aldrich (purity ≥95%, Co. St. Louis, MO, United States), as shown in Supplementary Table S2. All plant volatiles were diluted into 1 M with dimethyl sulfoxide (DMSO) as the stock solution and stored in −20 C until use. The stock solutions were diluted to 10^–4^ M with 1 × Ringer’s buffer (2 mM KCl, 0.8 mM CaCl_2_, 5 mM HEPES, 5 mM MgCl_2_, 96 mM NaCl, pH 7.6) for ligand screening of PxylOR11. In EAG experiments and behavioral assays, the three selected compounds, benzyl alcohol, salicylaldehyde and phenylacetaldehyde, were dissolved in hexane at different concentrations: 10, 100 and 1,000 ng/μL. The plant volatile samples used in each experiment were formulated freshly.

### 2.6 Vector Construction and cRNA Synthesis

Primers with restriction enzyme sites (BcuI and NotI), and Kozak consensus sequences were designed to amplify the full lengths of the PxylOR11 (Supplementary Table S1). The same restriction enzyme cutting sites (BcuI and NotI) were used in the expression vector (pT7Ts), and PxylOR11 was ligated into pT7Ts (Wang et al., 2010). The expression vector of PxylOrco has been constructed in our previous work (Liu et al., 2018). Combined with the sequencing results, the recombinant plasmid of PxylOR11 was extracted and linearized by SmaI. The mMESSAGE Mmachine T7 kit (Ambion, Austin, TX, United States) was used for the synthesis of cRNA. The cRNA of PxylOrco and PxylOR11 was diluted to 2 µg/µL with nuclease-free water and stored at −80 C.

### 2.7 Expression of PxylOR11 in *Xenopus* Oocytes and Two Electrode Voltage-Clamp Recordings

The methods of *Xenpous* Oocytes expression and two electrode volage-clamp recordings refer to the previously reported protocols (Lu et al., 2007; Wang et al., 2010). The diluted cRNA (PxylOrco and PxylOR11) was mixed with equal volume ratio, and 27.6 ng of each cRNA was injected into a mature healthy *Xenopus* oocyte (Wang et al., 2011). 1 × Ringer’s solution (100 mg/ml streptomycin, 5% dialyzed horse serum, 550 mg/ml sodium pyruvate and 55 mg/ml tetracycline) was used to incubated the injected oocytes for 3–4 days at 16 C. Plant volatile compounds induced currents were recorded with an OC-725C oocyte clamp (Warner Instruments, Hamden, CT, United States) at holding potential of −80 mV. Oocytes (PxylOR11/PxylOrco) were exposed to plant volatile compounds in ascending order of concentration with an interval between exposures that allowed the current to return to baseline. The acquire and analyze of two electrode voltage-clamp recording by the Digidata 1440A and pCLAMP 10.2 software (Axon Instrument Inc., Union City, CA, United States). GraphPad Prism 5 (GraphPad Software Inc., San Diego, CA, United States) was used to analyzed dose-response data.

### 2.8 Electroantennogram Recording

The antennae of *P. xylostella* adults (female and male moths) were cut off from the base, and tip segments were removed at the end. The glass electrode (KCl, 0.1 mol/L) was used to connect the antennae to the electroantennogram, which consists of an air stimulus controller (CS-55, Syntech, Hilversum, Netherlands), 10 × AC/DC Headstage Preamplifier (Syntech, Hilversum, Netherlands) and Intelligent Data Acquisition Controller (IDAC-4-USB, Syntech, Hilversum, Netherlands). The Syntech EAG software 2.0 was used to recorded (Syntech, Hilversum, Netherlands). Benzyl alcohol, salicylaldehyde, and phenylacetaldehyde were diluted in hexane at concentrations of 10, 100 and 1,000 ng/μL. Take 10 μL test solution on the filter paper strip (1 × 5 cm) and placed into a Pasteur pipette. The antenna was exposed to a constant charcoal-filtered humid air flow (500 ml/min) through a metal tube for a stimulation time of 0.2 s. The female and male moths were repeated 10 times each. EAG responses for each compound were calculated by EAG response to the test compound EAG response to the blank. Data analysis used mean ± standard error of the mean (SEM) and independent *t*-test.

### 2.9 Behavioral Assays

The 4th instar larvae of *P. xylostella* were divided into male and female and put into plastic boxes (20 × 13 × 7 cm) until eclosion. Two-day-old male and female moths were test in a Y-tube (15 cm base tube; 24.5 cm arms at a 45° angle; diameter, 2.5 cm) olfactory. Place the Y-tube inside the behavior box (100 × 60 × 80 cm) and install the fluorescent lamp to provide light. Air was pumped through Teflon tubing, purified by passage through activated charcoal and humidified by bubbling through distilled water. Airflow was divided and then passed through two separate flow meters, which regulated the flow rate to 400 ml/min. All behavioral experiments were conducted in a temperature-controlled room at 26 C. Based on the results of the two-electrode voltage-clamp and EAG recordings, we chose benzyl alcohol, salicylaldehyde and phenylacetaldehyde for behavioral assays. The three compounds were separately diluted in hexane at concentrations of 10, 100 and 1,000 ng/μL. Take 10 μL test solution on the filter paper strip (2 × 2 cm) and insert it into “treatment” arm. 10 μL hexane was placed in the “control” arm. One moth (female or male) was put into the base tube for 10 min. During this time, moths entered the treatment or control arms more than 5 cm, and stayed for at least 30 s was classified as “making a choice”. Moths did not move and remained at the base of the Y-tube were recorded as “no choice”. Each concentration of the test solution was repeated for 30 times. Data were collected from a total of 30 male moths and 30 female moths for benzyl alcohol, salicylaldehyde and phenylacetaldehyde. Before the experiment, the Y-tube was washed with ethanol, and then dried at 100 C for 2 h. Statistical analyses were performed by χ^2^ goodness-of-fit using the SPSS13.0 software.

## 3 Results

### 3.1 Gene Cloning and Phylogenetic Analysis

**FIGURE 1:**
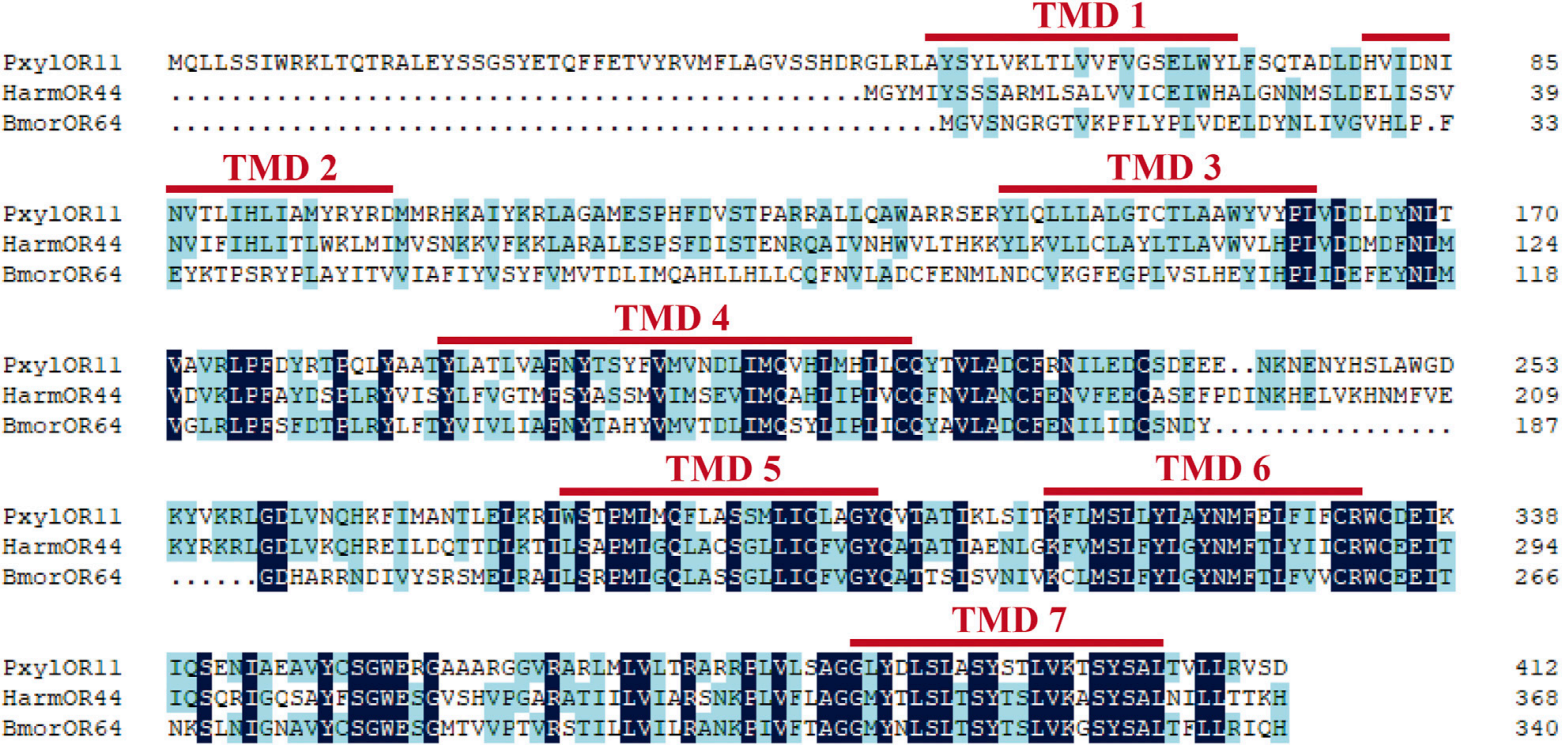
Amino acid alignment of PxylOR11 homologous genes. Pxyl, *P. xylostella*; Harm, *H. armigera*; Bmor, *B. mori*. The seven transmembrane domains (TM1-TM7) are marked with red lines at the top.

**FIGURE 2:**
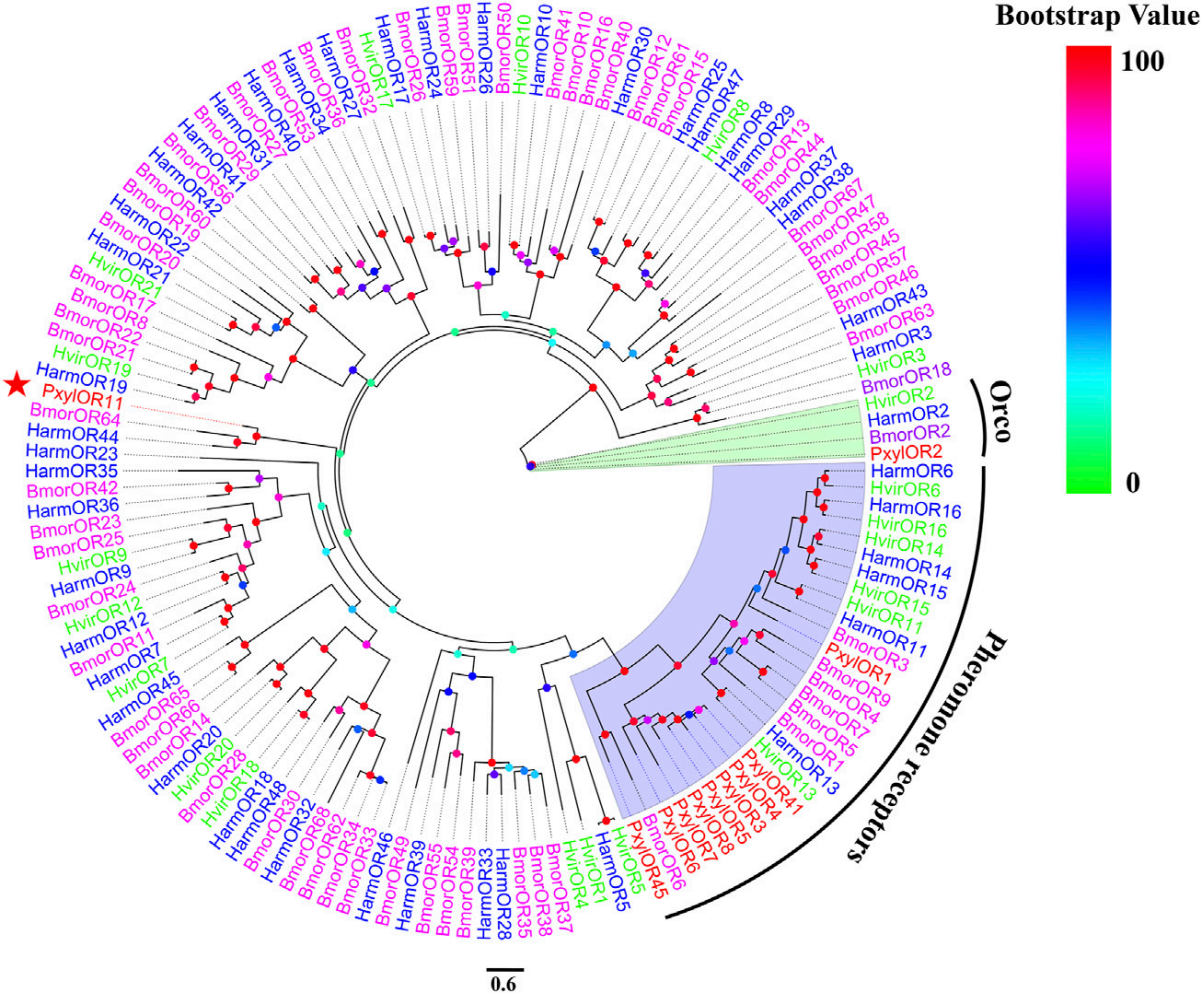
Phylogenetic analysis of PxylOR11 with candidate ORs from Lepidoptera. Pxyl, *P. xylostella* (red); Harm, *H. armigera* (blue); Hvir, *H. virescens* (green); Bmor, *B. mori* (purple). The clade in green indicates the Orco co-receptor gene clade and the one in blue is the PRs gene clade. The PxylOR11 is indicated with the red star.

Based on the previous research results after analyzing *P. xylostella* in the laboratory, we cloned the full-length ORF of the PxylOR11 (GenBank: OM937945) gene encoding a 413 amino acid protein from mixed cDNAs (male and female antennae). The transmembrane domain prediction results demonstrated that PxylOR11 has seven transmembrane domains (TMDs) (Figure 1), with the N-terminus inside and C-terminus outside. Two amino acid sequences from *B. mori* (BmorOR64) and *H. armigera* (HarmOR44) were aligned with PxylOR11 and found to have 32 and 41% identity, respectively (Figure 1). To clarify the evolutionary relationship between PxylOR11 and the ORs of other Lepidoptera insects, the phylogenetic tree of *B. mori*, *H. armigera*, *H. virescens*, *P. xylostella* PRs and Orco was constructed (Figure 2). The phylogenetic tree showed that all Orco and PRs can be clustered in different branches. PxylOR11 was clustered with Lepidoptera other general ORs and was separate from PRs, indicating that PxylOR11 belongs to general ORs of *P. xylostella*.

### 3.2 PxylOR11 is Specifically Expressed in Antennae

**FIGURE 3:**
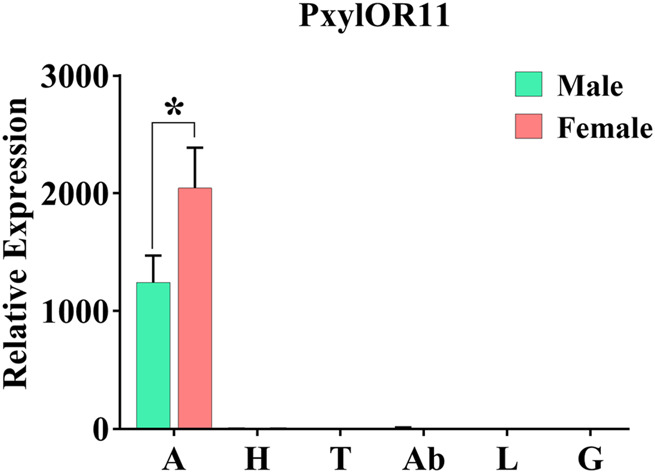
Expression profiles of PxylOR11 in different tissues of female and male *P. xylostella*. A, antennae; H, heads; T, thoraxes; Ab, abdomens; L, legs; G, genitalia. The one asterisks indicate significant differences between males and females (*t*-Test, *p* < 0.05). Error bars indicate the SEM (*n* = 3).

We performed real-time quantitative polymerase chain reaction (RT-qPCR) to evaluate the expression levels of the PxylOR11 gene in different tissues of male and female *P. xylostella*. The results demonstrated that PxylOR11 was specifically expressed in the antennae, and its expression was difficult to detect in other tissues including the head (without antennae), leg, genital, thorax and abdomen. The expression levels of PxylOR11 in female antennae were significantly higher than in male antennae (*p* < 0.05) (Figure 3).

### 3.3 PxylOR11 Finely Tunes to Three Aromatic Volatiles

**FIGURE 4:**
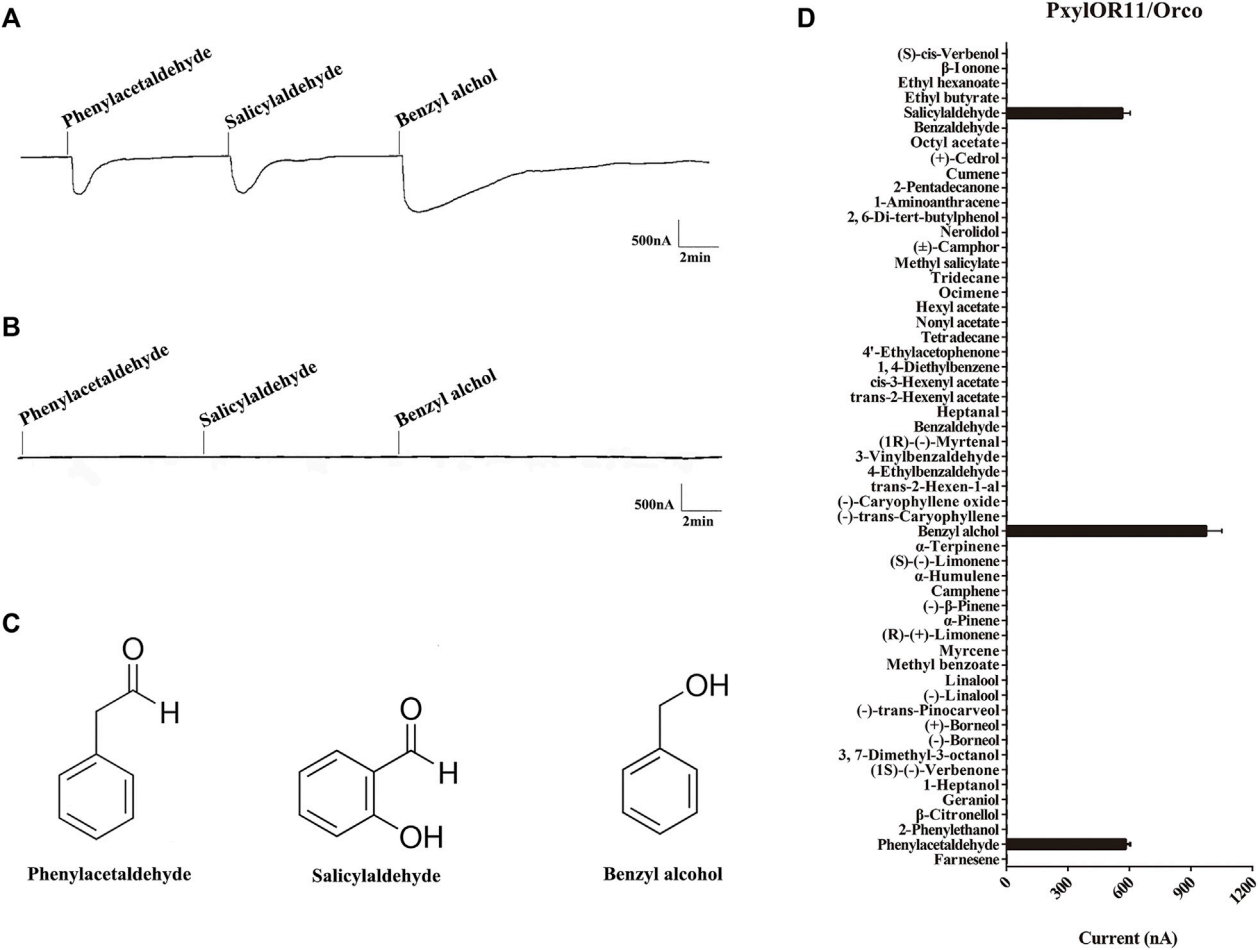
Responses of *P. xylostella* OR11/Orco to plant volatile compounds by *in vitro* functional scanning. **(A)** Inward current responses of PxylOR11/PxylOrco *Xenopus* oocytes exposed to compounds. **(B)** Buffer-injected *Xenopus* oocytes failed to respond to any of the compounds. **(C)** The structures of three aromatic compounds benzyl alcohol, salicylaldehyde, and phenylacetaldehyde are shown. **(D)** Response profile of PxylOR11/PxylOrco *Xenopus* oocytes. Error bar indicate SEM (*n* = 6).

The voltage clamp system was used to record the functional characterization of PxylOR11, which was co-expressed with PxylOrco in *Xenopus* oocytes. Of all 55 plant volatile compounds, 15 aromatic compounds were included. Our results show that PxylOR11 can be activated by 3 of 55 plant volatile compounds at a concentration of 10^–4^ (Figure 4A). We recorded strong responses to benzyl alcohol, with an average value of 975 nA (*n* = 7, Figure 4D). Lower intensity signals were measured with phenylacetaldehyde and salicylaldehyde, with average currents of 582 and 565 nA, respectively (*n* = 6, Figure 4D). As a control, oocytes injected with the buffer did not respond to the above plant volatiles (Figure 4B).

### 3.4 Electrophysiological Responses of *P. xylostella* Antennae to Three Aromatic Volatiles

**FIGURE 5:**
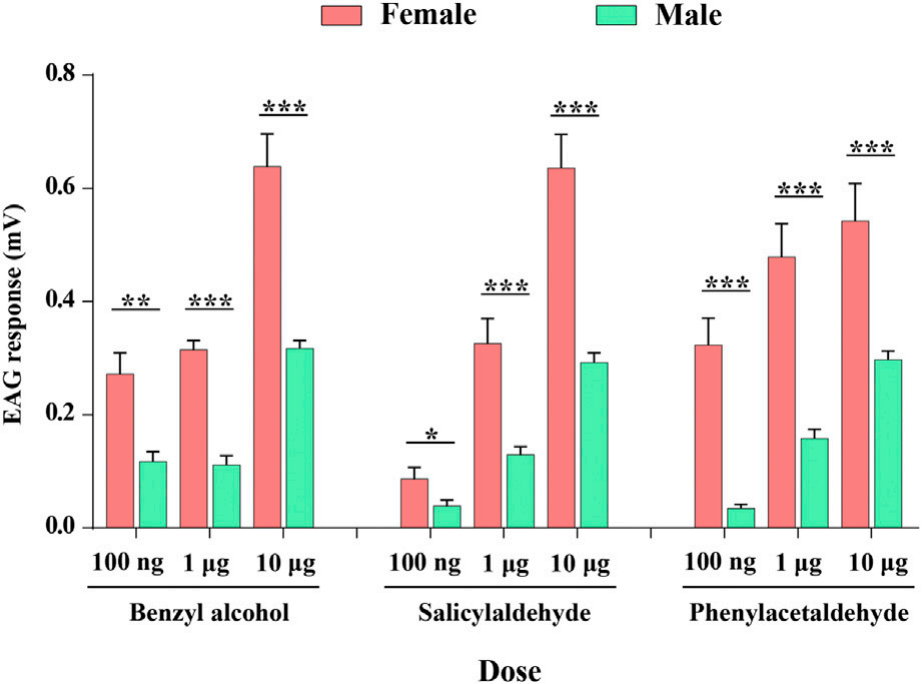
Electrophysiological responses measured as electroantennograms (EAG) of female and male *P. xylostella* antennae to three ligands (benzyl alcohol, salicylaldehyde, and phenylacetaldehyde) of PxylOR11. Error bars indicate SEM (*n* = 10). Asterisks indicate the significant differences between females and males. *, *p* < 0.05; **, *p* < 0.01; ***, *p* < 0.001.

We recorded the electrophysiological responses of male and female of *P. xylostella* antennae to three compounds (benzyl alcohol, salicylaldehyde, and phenylacetaldehyde) of ligands of PxylOR11 in the dose-response experiments. Female and male antennae presented a dose-dependent EAG response to all compounds, with responses increasing as compound doses increased, except for benzyl alcohol. At the maximum dose (10 μg), the EAG response values to benzyl alcohol, salicylaldehyde, and phenylacetaldehyde were highest, with mean response values of 0.64 and 0.31 mV, 0.63 and 0.29 mV, 0.54 mV, and 0.29 mV, for females and males, respectively. At all doses, the female EAG response values were significantly higher than that of the males (Figure 5).

### 3.5 Repellent Effect of the Three Aromatic Volatiles on *P. xylostella*

**FIGURE 6:**
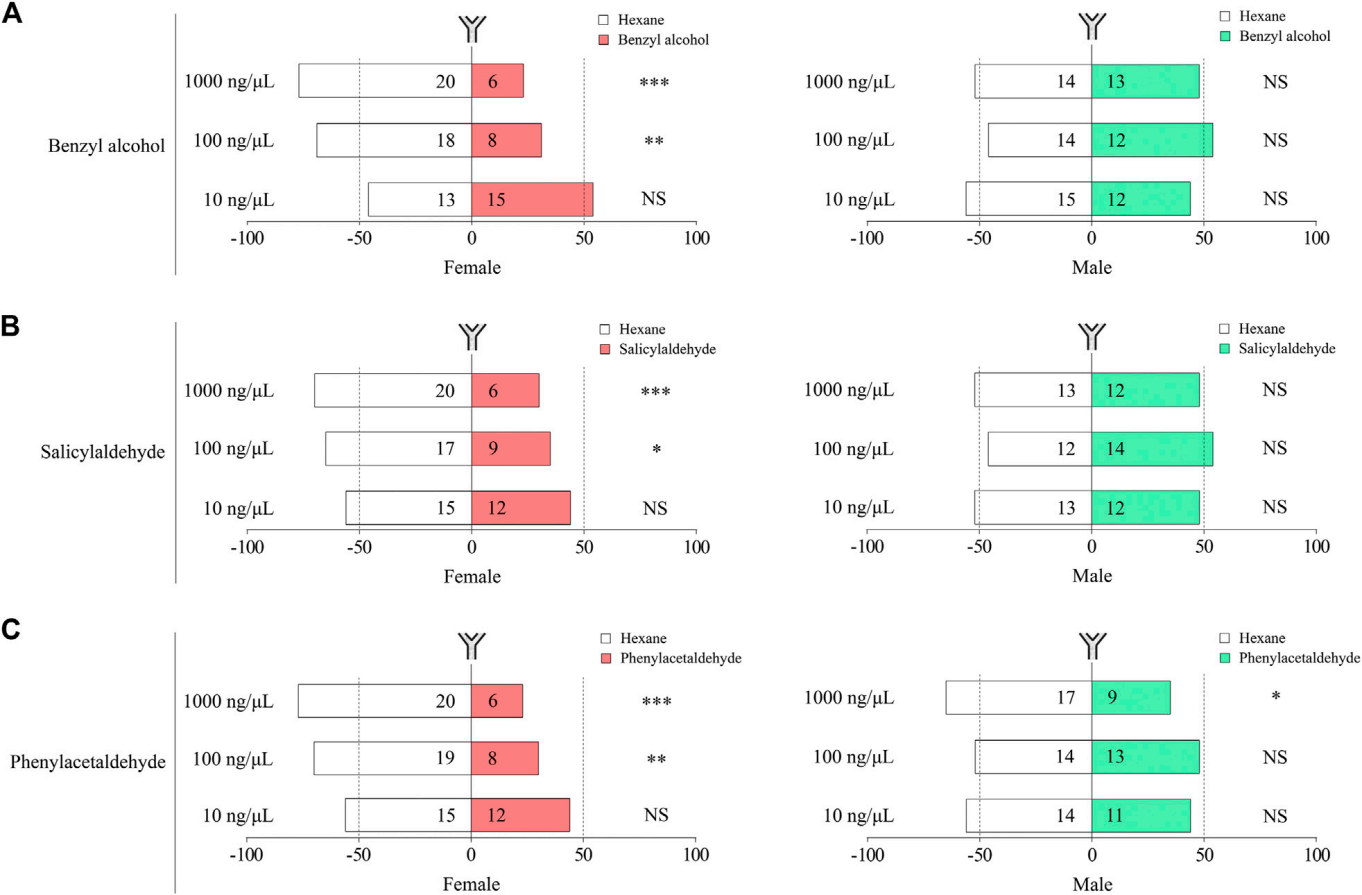
Behavioral responses of female and male *P. xylostella* to benzyl alcohol **(A)**, salicylaldehyde **(B)**, and phenylacetaldehyde **(C)**. Each bar shows % of *P. xylostella* that chose either of the odor sources. Numbers in bars are the total numbers of *P. xylostella* choosing that odor sources. Choices between odor sources were analyzed with χ^2^ goodness-of-fit tests on numbers (NS, no significant difference, *p* > 0.05; ***p < 0.001; **, *p* < 0.01; *, *p* < 0.05).

We further tested the effects of benzyl alcohol, salicylaldehyde, and phenylacetaldehyde on the female and male moths of *P. xylostella* using the Y-tube assays. Our results demonstrate that benzyl alcohol, salicylaldehyde, and phenylacetaldehyde had a significant repellent effect on female moths at concentrations of 100 and 1,000 ng/μL (Figure 6). Under similar conditions, male moths had no behavioral regulation effect to all concentrations of benzyl alcohol, salicylaldehyde, and phenylacetaldehyde, except for phenylacetaldehyde at a concentration of 1,000 ng/μL, which had a significant repellent effect on male moths (Figure 6). *P. xylostella* behavior was affected by sex and concentrations of benzyl alcohol, salicylaldehyde and phenylacetaldehyde.

## 4 Discussion

Olfaction is the main sensory modality in insects, and plays an important role when the insect is searching for host plants, choosing mates and oviposition sites, and evading predators. Based on innate characteristics, we can research and develop repellent, attractant, or mating interfering agents that can be used in pest control. In recent years, the reverse chemical ecology approach has been widely used to screen insect behavior (Choo et al., 2018), opening up innovative perspectives in OR-based screening of potential behavioral regulators (repellents and attractants) for their application in pest management. In this study, based on the transcriptome analysis of *P. xylostella* (Yang et al., 2017), we identified PxylOR11 from the antennae of *P. xylostella*, and obtained the full-length sequences, and studied the binding selectivity of PxylOR11.

qRT-PCR analysis revealed that PxylOR11 was specifically expressed in the antennae, and that the expression levels in female antennae were significantly higher than in male antennae. OfurOR27 is also highly expressed in the antennae of female *O. furnacalis* and participates in the identification of oviposition repellents (Yu et al., 2020). We hypothesized that PxylOR11 is also involved in the identification of some important volatiles (repellents and attractants) by *P. xylostella*, so we used a reverse chemical ecology approach to identify PxylOR11 ligands and found that PxylOR11 was activated by three aromatic compounds (benzyl alcohol, phenylacetaldehyde, and salicylaldehyde). Aromatic compounds belong to a large group of plant volatiles and are important to insects (Tabanca et al., 2013; Knauer and Schiestl, 2015). The functional evolutionary analysis of ORs showed that most ORs that recognize aromatic compounds were differentiated first and that their functions were relatively conserved (Guo et al., 2020). Overall, this suggests that PxylOR11 plays an important role in the recognition of aromatic compounds by *P. xylostella*.

Plants produce volatiles during growth and development, which are composed of multiple components and play an important role in the feeding, oviposition and host positioning of herbivorous insects (Baldwin, 2010). When plants are attacked by herbivores, they produce herbivore induced plant volatiles (HIPVs) that differ from healthy plant volatiles in a species-specific manner. HIPVs have multiple functions, including repelling herbivorous insects, attracting the natural enemies of herbivores, and regulating the interactions between plants (Baldwin et al., 2006; Unsicker et al., 2009; Li et al., 2018). For herbivores, HIPVs indicate that there are already competitors or predators on the host plants. Herbivores typically avoid these damaged plants because of their low quality, the direct defense response initiated by the host plants, or the presence of natural enemies (Turlings and Erb, 2018). The conventional chemical ecology approach has found that multiple HIPVs have repellent effects on a variety of herbivores (Birkett et al., 2000). When *Nicotiana attenuata* is attacked by herbivores (*Epitrix hirtipennis*, *Manduca quinquemaculata* and *Dicyphus minimus*), HIPVs can increase egg predation rates by a generalist predator (Kessler and Baldwin, 2001). Similarly, cruciferous plants harmed by *P. xylostella* will release HIPVs, which can significantly attract parasitoids (*Cotesia vestalis*) of *P. xylostella* larvae (Shiojiri et al., 2010). HIPVs can cause significant behavioral changes in different community members, from carnivorous insects to natural enemies, all of which will have different responses under the influence of HIPVs (Dicke and Baldwin, 2010).

The three aromatic compounds of PxylOR11 ligands also belong to HIPVs of cruciferous plants (Fernandes et al., 2010; Pierre et al., 2011; Yang et al., 2016). Benzyl alcohol has different functions, depending on the species. In our study, we found that benzyl alcohol had a repellent effect on female *P. xylostella*. Similarly, the *Aesculus hippocastanum* damaged by the *Cameraria ohridella* can also release benzyl alcohol, which has an oviposition inhibitory effect on *C. ohridella* (Johne et al., 2006). For other herbivores, benzyl alcohol has an attractive effect, for example, *Epiphyas postvittana* and *Ectropis obliqua* are attracted to benzyl alcohol released (HIPVs) by host plants (Sun et al., 2014; El-Sayed et al., 2016). For natural enemies, benzyl alcohol has an obvious attraction effect on *C. vestalis*, which is an endoparasitoid wasp that attacks *P. xylostella* larvae (Yang et al., 2016). Similarly, we found that salicylaldehyde has a significant repellent effect on female *P. xylostella*. The repellent effect of salicylaldehyde also occurs in *Frankliniella occidentalis* (Koschier et al., 2007), while salicylaldehyde is known as a defensive agent (e.g., larval defensive secretions of chrysomelid beetles against their natural enemies, the main component of which is salicylaldehyde) (Pasteels and Grégoire, 1983). Phenylacetaldehyde is different from the above two compounds in that it has a repellent effect on both sexes of *P. xylostella*. This repellent effect of phenylacetaldehyde also exists in *Apis florea* (Naik et al., 2010) Additionally, compounds such as phenylacetaldehyde, which has repellent effects on both male and female insects, also include myrtenol (*Dendroctonus armandi*) and hexanal (*Holotrichia parallela*) (Wang X. et al., 2020; Zhao et al., 2020). Adult herbivores are repelled by host plants infested with larvae, while others are attracted by infested plants (Rojas, 1999; De Moraes et al., 2001; Signoretti et al., 2011; Sun et al., 2014). These same compounds induce different behavioral responses in different herbivores. For example, nonanal has an oviposition repellent effect on *O. furnacalis*, but an oviposition attractant effect on *H. assulta* (Wang C. et al., 2020; Yu et al., 2020). All these suggest that benzyl alcohol, salicylaldehyde, and phenylacetaldehyde may play important roles in the interaction between host plants, *P. xylostella*, and natural enemies.

In the *In vitro* functional studies, PxylOR11 was tuned to three aromatic compounds in our odor panel, and all three aromatic compounds have a repellent effect on *P. xylostella*. The repellent effect of all ligands from one OR also occurred in *O. furnacalis* (OfurOR27) (Yu et al., 2020). However, compared with benzyl alcohol, PxylOR11 has a relatively small electrophysiological response to phenylacetaldehyde and salicylaldehyde, indicating that there could be other PxylORs that have a strong response to these two compounds. Studies have demonstrated that a single OR can recognize multiple compounds, while multiple ORs can also recognize one compound (Cui et al., 2018). *P. xylostella* uses HIPVs released by host plans to locate oviposition sites, which also occurs in the *Spodoptera frugiperda* (Yactayo-Chang et al., 2021). This suggests that moths use HIPVs to identify and avoid infested plants.

In summary, plants will release benzyl alcohol, salicylaldehyde, and phenylacetaldehyde for self-defense after being harmed by *P. xylostella*. Benzyl alcohol is a HIPV for host plants, a repellent for *P. xylostella*, and an attractant for natural enemies. Research on how herbivores recognize HIPVs will benefit the development of a push-pull system and pest control based on chemical ecology (Pickett et al., 2014). Using reverse chemical ecology, we screened three aromatic compounds that have a repellent effect on *P. xylostella*, and studied the recognition mechanism of three aromatic compounds by *P. xylostella* at the molecular level. Our research will help the development of *P. xylostella* repellent, which can provide novel methods for the integrated control of *P. xylostella*.

## Data Availability

The original contributions presented in the study are included in the article/Supplementary Materials, further inquiries can be directed to the corresponding authors.

## References

[B1] Ache B. W. Kennedy C. E. J. (1987). Insect Chemoreception: Mechanisms in Insect Olfaction. Science 236, 341. 10.1126/science.236.4799.341 17755559

[B2] Baldwin I. T. Halitschke R. Paschold A. Von Dahl C. C. Preston C. A. (2006). Volatile Signaling in Plant-Plant Interactions: "Talking Trees" in the Genomics Era. Science 311, 812–815. 10.1126/science.1118446 16469918

[B3] Baldwin I. T. (2010). Plant Volatiles. Curr. Biol. 20, R392–R397. 10.1016/j.cub.2010.02.052 20462477

[B4] Birkett M. A. Campbell C. A. M. Chamberlain K. Guerrieri E. Hick A. J. Martin J. L. (2000). New Roles for Cis -jasmone as an Insect Semiochemical and in Plant Defense. Proc. Natl. Acad. Sci. U.S.A. 97, 9329–9334. 10.1073/pnas.160241697 10900270 PMC16867

[B5] Cao D. Liu Y. Wei J. Liao X. Walker W. B. Li J. (2014). Identification of Candidate Olfactory Genes in *Chilo Suppressalis* by Antennal Transcriptome Analysis. Int. J. Biol. Sci. 10, 846–860. 10.7150/ijbs.9297 25076861 PMC4115196

[B6] Choo Y.-M. Xu P. Hwang J. K. Zeng F. Tan K. Bhagavathy G. (2018). Reverse Chemical Ecology Approach for the Identification of an Oviposition Attractant for *Culex quinquefasciatus* . Proc. Natl. Acad. Sci. U.S.A. 115, 714–719. 10.1073/pnas.1718284115 29311316 PMC5789951

[B7] Clyne P. J. Warr C. G. Freeman M. R. Lessing D. Kim J. Carlson J. R. (1999). A Novel Family of Divergent Seven-Transmembrane Proteins. Neuron 22, 327–338. 10.1016/s0896-6273(00)81093-4 10069338

[B8] Cui W.-c. Wang B. Guo M.-b. Liu Y. Jacquin-Joly E. Yan S.-c. (2018). A Receptor-Neuron Correlate for the Detection of Attractive Plant Volatiles in *Helicoverpa Assulta* (Lepidoptera: Noctuidae). Insect Biochem. Mol. Biol. 97, 31–39. 10.1016/j.ibmb.2018.04.006 29698698

[B9] de Bruyne M. Baker T. C. (2008). Odor Detection in Insects: Volatile Codes. J. Chem. Ecol. 34, 882–897. 10.1007/s10886-008-9485-4 18535862

[B10] de Fouchier A. Walker W. B. Montagné N. Steiner C. Binyameen M. Schlyter F. (2017). Functional Evolution of Lepidoptera Olfactory Receptors Revealed by Deorphanization of a Moth Repertoire. Nat. Commun. 8, 15709. 10.1038/ncomms15709 28580965 PMC5465368

[B11] De Moraes C. M. Mescher M. C. Tumlinson J. H. (2001). Caterpillar-induced Nocturnal Plant Volatiles Repel Conspecific Females. Nature 410, 577–580. 10.1038/35069058 11279494

[B12] Dicke M. Baldwin I. T. (2010). The Evolutionary Context for Herbivore-Induced Plant Volatiles: beyond the 'cry for Help'. Trends Plant Sci. 15, 167–175. 10.1016/j.tplants.2009.12.002 20047849

[B13] Du L. Zhao X. Liang X. Gao X. Liu Y. Wang G. (2018). Identification of Candidate Chemosensory Genes in *Mythimna Separata* by Transcriptomic Analysis. BMC Genomics 19, 518. 10.1186/s12864-018-4898-0 29973137 PMC6030794

[B14] El-Sayed A. M. Knight A. L. Byers J. A. Judd G. J. R. Suckling D. M. (2016). Caterpillar-induced Plant Volatiles Attract Conspecific Adults in Nature. Sci. Rep. 6, 37555. 10.1038/srep37555 27892474 PMC5124949

[B15] Fernandes F. Pereira D. M. Guedes de Pinho P. Valentão P. Pereira J. A. Bento A. (2010). Headspace Solid-phase Microextraction and Gas Chromatography/ion Trap-Mass Spectrometry Applied to a Living System: Pieris Brassicae Fed with Kale. Food Chem. 119, 1681–1693. 10.1016/j.foodchem.2009.09.046

[B16] Furlong M. J. Wright D. J. Dosdall L. M. (2013). Diamondback Moth Ecology and Management: Problems, Progress, and Prospects. Annu. Rev. Entomol. 58, 517–541. 10.1146/annurev-ento-120811-153605 23020617

[B17] Gao Q. Chess A. (1999). Identification of Candidate *Drosophila* Olfactory Receptors from Genomic DNA Sequence. Genomics 60, 31–39. 10.1006/geno.1999.5894 10458908

[B18] Guo H. Gong X. L. Li G. C. Mo B. T. Jiang N. J. Huang L. Q. (2022). Functional Analysis of Pheromone Receptor Repertoire in the Fall Armyworm, *Spodoptera Frugiperda* . Pest Manag. Sci. 78, 2052–2064. 10.1002/ps.6831 35124874

[B19] Guo M. Du L. Chen Q. Feng Y. Zhang J. Zhang X. (2020). Odorant Receptors for Detecting Flowering Plant Cues Are Functionally Conserved across Moths and Butterflies. Mol. Biol. Evol. 38, 1413–1427. 10.1093/molbev/msaa300 PMC8042770 33231630

[B20] Johne A. B. Weissbecker B. Schütz S. (2006). Volatile Emissions from *Aesculus Hippocastanum* Induced by Mining of Larval Stages of *Cameraria Ohridella* Influence Oviposition by Conspecific Females. J. Chem. Ecol. 32, 2303–2319. 10.1007/s10886-006-9146-4 17001531

[B21] Kessler A. Baldwin I. T. (2001). Defensive Function of Herbivore-Induced Plant Volatile Emissions in Nature. Science 291, 2141–2144. 10.1126/science.291.5511.2141 11251117

[B22] Knauer A. C. Schiestl F. P. (2015). Bees Use Honest Floral Signals as Indicators of Reward when Visiting Flowers. Ecol. Lett. 18, 135–143. 10.1111/ele.12386 25491788

[B23] Koschier E. H. Hoffmann D. Riefler J. (2007). Influence of Salicylaldehyde and Methyl Salicylate on Post-landing Behaviour of *Frankliniella Occidentalis* Pergande. J. Appl. Entomol. 131, 362–367. 10.1111/j.1439-0418.2007.01191.x

[B24] Krieger J. Grosse-Wilde E. Gohl T. Dewer Y. M. E. Raming K. Breer H. (2004). Genes Encoding Candidate Pheromone Receptors in a Moth ( *Heliothis virescens* ). Proc. Natl. Acad. Sci. U.S.A. 101, 11845–11850. 10.1073/pnas.0403052101 15289611 PMC511062

[B25] Larsson M. C. Domingos A. I. Jones W. D. Chiappe M. E. Amrein H. Vosshall L. B. (2004). Or83b Encodes a Broadly Expressed Odorant Receptor Essential for *Drosophila* Olfaction. Neuron 43, 703–714. 10.1016/j.neuron.2004.08.019 15339651

[B26] Leal W. S. (2013). Odorant Reception in Insects: Roles of Receptors, Binding Proteins, and Degrading Enzymes. Annu. Rev. Entomol. 58, 373–391. 10.1146/annurev-ento-120811-153635 23020622

[B27] Lecomte C. Pierre D. Pouzat J. Thibout E. (1998). Behavioural and Olfactory Variations in the Leek Moth, *Acrolepiopsis Assectella*, after Several Generations of Rearing under Diverse Conditions. Entomologia Exp. Appl. 86, 305–311. 10.1046/j.1570-7458.1998.00293.x

[B28] Li F. Li W. Lin Y.-J. Pickett J. A. Birkett M. A. Wu K. (2018). Expression of lima Bean Terpene Synthases in Rice Enhances Recruitment of a Beneficial Enemy of a Major Rice Pest. Plant Cell Environ. 41, 111–120. 10.1111/pce.12959 28370092

[B29] Li H.-M. Liu W.-B. Yang L.-L. Cao H.-Q. Pelosi P. Wang G.-R. (2020). Aromatic Volatiles and Odorant Receptor 25 Mediate Attraction of *Eupeodes Corollae* to Flowers. J. Agric. Food Chem. 68, 12212–12220. 10.1021/acs.jafc.0c03854 33103425

[B30] Liu C. Liu Y. Guo M. Cao D. Dong S. Wang G. (2014). Narrow Tuning of an Odorant Receptor to Plant Volatiles inSpodoptera exigua(Hübner). Insect Mol. Biol. 23, 487–496. 10.1111/imb.12096 24779920

[B31] Liu C. Liu Y. Walker W. B. Dong S. Wang G. (2013). Identification and Functional Characterization of Sex Pheromone Receptors in Beet Armyworm Spodoptera Exigua (Hübner). Insect Biochem. Mol. Biol. 43, 747–754. 10.1016/j.ibmb.2013.05.009 23751753

[B32] Liu X.-L. Zhang J. Yan Q. Miao C.-L. Han W.-K. Hou W. (2020). The Molecular Basis of Host Selection in a Crucifer-Specialized Moth. Curr. Biol. 30, 4476–4482. 10.1016/j.cub.2020.08.047 32916118

[B33] Liu Y. Gu S. Zhang Y. Guo Y. Wang G. (2012). Candidate Olfaction Genes Identified within the *Helicoverpa Armigera* Antennal Transcriptome. Plos One 7, e48260. 10.1371/journal.pone.0048260 23110222 PMC3482190

[B34] Liu Y. Liu Y. Jiang X. Wang G. (2018). Cloning and Functional Characterization of Three New Pheromone Receptors from the Diamondback Moth, Plutella Xylostella. J. Insect Physiology 107, 14–22. 10.1016/j.jinsphys.2018.02.005 29438663

[B35] Livak K. J. Schmittgen T. D. (2001). Analysis of Relative Gene Expression Data Using Real-Time Quantitative PCR and the 2−ΔΔCT Method. Methods 25, 402–408. 10.1006/meth.2001.1262 11846609

[B36] Lu T. Qiu Y. T. Wang G. Kwon J. Y. Rutzler M. Kwon H.-W. (2007). Odor Coding in the Maxillary Palp of the Malaria Vector Mosquito *Anopheles gambiae* . Curr. Biol. 17, 1533–1544. 10.1016/j.cub.2007.07.062 17764944 PMC3113458

[B37] Naik D. G. Puntambekar H. Anantpure P. (2010). Essential Oil of *Terminalia Chebula* Fruits as a Repellent for the Indian Honeybee *Apis Florea* . Chem. Biodivers. 7, 1303–1310. 10.1002/cbdv.200900274 20491085

[B38] Pasteels J. M. Grégoire J. C. Rowell-Rahier M. (1983). The Chemical Ecology of Defense in Arthropods. Annu. Rev. Entomol. 28, 263–289. 10.1146/annurev.en.28.010183.001403

[B39] Pettersson J. Karunaratne S. Ahmed E. Kumar V. (1998). The Cowpea Aphid, *Aphis Craccivora*, Host Plant Odours and Pheromones. Entomologia Exp. Appl. 88, 177–184. 10.1046/j.1570-7458.1998.00360.x

[B40] Pickett J. A. Woodcock C. M. Midega C. A. Khan Z. R. (2014). Push-pull Farming Systems. Curr. Opin. Biotechnol. 26, 125–132. 10.1016/j.copbio.2013.12.006 24445079

[B41] Pierre P. S. Jansen J. J. Hordijk C. A. van Dam N. M. Cortesero A.-M. Dugravot S. (2011). Differences in Volatile Profiles of Turnip Plants Subjected to Single and Dual Herbivory above- and Belowground. J. Chem. Ecol. 37, 368–377. 10.1007/s10886-011-9934-3 21448706 PMC3197925

[B42] Rojas J. C. (1999). Influence of Host Plant Damage on the Host-Finding Behavior ofMamestra brassicae(Lepidoptera: Noctuidae). Environ. Entomol. 28, 588–593. 10.1093/ee/28.4.588

[B43] Shiojiri K. Ozawa R. Kugimiya S. Uefune M. van Wijk M. Sabelis M. W. (2010). Herbivore-specific, Density-dependent Induction of Plant Volatiles: Honest or "cry Wolf" Signals? PLoS One 5, e12161. 10.1371/journal.pone.0012161 20808961 PMC2923144

[B44] Signoretti A. G. C. Peñaflor M. F. G. V. Moreira L. S. D. Noronha N. C. Bento J. M. S. (2011). Diurnal and Nocturnal Herbivore Induction on Maize Elicit Different Innate Response of the Fall Armyworm Parasitoid, *Campoletis Flavicincta* . J. Pest Sci. 85, 101–107. 10.1007/s10340-011-0397-7

[B45] Suh E. Bohbot J. D. Zwiebel L. J. (2014). Peripheral Olfactory Signaling in Insects. Curr. Opin. Insect Sci. 6, 86–92. 10.1016/j.cois.2014.10.006 25584200 PMC4288021

[B46] Sun M. Liu Y. Walker W. B. Liu C. Lin K. Gu S. (2013). Identification and Characterization of Pheromone Receptors and Interplay between Receptors and Pheromone Binding Proteins in the Diamondback Moth, *Plutella Xyllostella* . PLoS One 8, e62098. 10.1371/journal.pone.0062098 23626773 PMC3633919

[B47] Sun X.-L. Wang G.-C. Gao Y. Zhang X.-Z. Xin Z.-J. Chen Z.-M. (2014). Volatiles Emitted from Tea Plants Infested by *Ectropis Obliqua* Larvae Are Attractive to Conspecific Moths. J. Chem. Ecol. 40, 1080–1089. 10.1007/s10886-014-0502-5 25378120

[B48] Tabanca N. Bernier U. R. Ali A. Wang M. Demirci B. Blythe E. K. (2013). Bioassay-guided Investigation of Two Monarda Essential Oils as Repellents of Yellow Fever Mosquito *Aedes aegypti* . J. Agric. Food Chem. 61, 8573–8580. 10.1021/jf402182h 23919579

[B49] Tamura K. Stecher G. Peterson D. Filipski A. Kumar S. (2013). MEGA6: Molecular Evolutionary Genetics Analysis Version 6.0. Mol. Biol. Evol. 30, 2725–2729. 10.1093/molbev/mst197 24132122 PMC3840312

[B50] Tanaka K. Uda Y. Ono Y. Nakagawa T. Suwa M. Yamaoka R. (2009). Highly Selective Tuning of a Silkworm Olfactory Receptor to a Key Mulberry Leaf Volatile. Curr. Biol. 19, 881–890. 10.1016/j.cub.2011.03.04610.1016/j.cub.2009.04.035 19427209

[B51] Turlings T. C. J. Erb M. (2018). Tritrophic Interactions Mediated by Herbivore-Induced Plant Volatiles: Mechanisms, Ecological Relevance, and Application Potential. Annu. Rev. Entomol. 63, 433–452. 10.1146/annurev-ento-020117-043507 29324043

[B52] Unsicker S. B. Kunert G. Gershenzon J. (2009). Protective Perfumes: the Role of Vegetative Volatiles in Plant Defense against Herbivores. Curr. Opin. Plant Biol. 12, 479–485. 10.1016/j.pbi.2009.04.001 19467919

[B53] Wang C. Li G. Miao C. Zhao M. Wang B. Guo X. (2020a). Nonanal Modulates Oviposition Preference in Female *Helicoverpa Assulta* (Lepidoptera: Noctuidae) via the Activation of Peripheral Neurons. Pest Manag. Sci. 76, 3159–3167. 10.1002/ps.5870 32333521 PMC7496960

[B54] Wang G. Carey A. F. Carlson J. R. Zwiebel L. J. (2010). Molecular Basis of Odor Coding in the Malaria Vector Mosquito *Anopheles gambiae* . Proc. Natl. Acad. Sci. U.S.A. 107, 4418–4423. 10.1073/pnas.0913392107 20160092 PMC2840125

[B55] Wang G. Vásquez G. M. Schal C. Zwiebel L. J. Gould F. (2011). Functional Characterization of Pheromone Receptors in the Tobacco Budworm *Heliothis virescens* . Insect Mol. Biol. 20, 125–133. 10.1111/j.1365-2583.2010.01045.x 20946532

[B56] Wang X. Wang S. Yi J. Li Y. Liu J. Wang J. (2020b). Three Host Plant Volatiles, Hexanal, Lauric Acid, and Tetradecane, Are Detected by an Antenna-Biased Expressed Odorant Receptor 27 in the Dark Black Chafer *Holotrichia Parallela* . J. Agric. Food Chem. 68, 7316–7323. 10.1021/acs.jafc.0c00333 32551589

[B57] Yactayo-Chang J. P. Mendoza J. Willms S. D. Rering C. C. Beck J. J. Block A. K. (2021). Zea mays Volatiles that Influence Oviposition and Feeding Behaviors of *Spodoptera Frugiperda* . J. Chem. Ecol. 47, 799–809. 10.1007/s10886-021-01302-w 34347233

[B58] Yang B. Ozaki K. Ishikawa Y. Matsuo T. (2015). Identification of Candidate Odorant Receptors in Asian Corn Borer *Ostrinia Furnacalis* . Plos One 10, e0121261. 10.1371/journal.pone.0121261 25803580 PMC4372370

[B59] Yang G. Zhang Y.-N. Gurr G. M. Vasseur L. You M.-S. (2016). Electroantennogram and Behavioral Responses ofCotesia Plutellaeto Plant Volatiles. Insect Sci. 23, 245–252. 10.1111/1744-7917.12308 26711914

[B60] Yang S. Cao D. Wang G. Liu Y. (2017). Identification of Genes Involved in Chemoreception in *Plutella Xyllostella* by Antennal Transcriptome Analysis. Sci. Rep. 7, 11941. 10.1038/s41598-017-11646-7 28931846 PMC5607341

[B61] You M. Ke F. You S. Wu Z. Liu Q. He W. (2020). Variation Among 532 Genomes Unveils the Origin and Evolutionary History of a Global Insect Herbivore. Nat. Commun. 11, 2321. 10.1038/s41467-020-16178-9 32385305 PMC7211002

[B62] Yu J. Yang B. Chang Y. Zhang Y. Wang G. (2020). Identification of a General Odorant Receptor for Repellents in the Asian Corn Borer *Ostrinia Furnacalis* . Front. Physiol. 11, 176. 10.3389/fphys.2020.00176 32231586 PMC7083148

[B63] Zalucki M. P. Shabbir A. Silva R. Adamson D. Shu-Sheng L. Furlong M. J. (2012). Estimating the Economic Cost of One of the World's Major Insect Pests, Plutella Xylostella (Lepidoptera: Plutellidae): Just How Long Is a Piece of String? Jnl. Econ. Entom. 105, 1115–1129. 10.1603/EC12107 22928287

[B64] Zhang R. Wang B. Grossi G. Falabella P. Liu Y. Yan S. (2017). Molecular Basis of Alarm Pheromone Detection in Aphids. Curr. Biol. 27, 55–61. 10.1016/j.cub.2016.10.013 27916525

[B65] Zhang Z. Zhang M. Yan S. Wang G. Liu Y. (2016). A Female-Biased Odorant Receptor from Apolygus Lucorum (Meyer-Dür) Tuned to Some Plant Odors. Ijms 17, 1165. 10.3390/ijms17081165 PMC5000588 27483241

[B66] Zhao M. Liu B. Sun Y. Wang Y. Dai L. Chen H. (2020). Presence and Roles of Myrtenol, Myrtanol and Myrtenal in D*endroctonus Armandi* (Coleoptera: Curculionidae: Scolytinae) and *Pinus Armandi* (Pinales: Pinaceae: Pinoideae). Pest. Manag. Sci. 76, 188–197. 10.1002/ps.5492 31106502

