# Supplementary material for: Odorant Receptor PxylOR11 Mediates Repellency of Plutella xylostella to Aromatic Volatiles
Source: Front Physiol. 2022 Jul 13;13:938555. doi: 10.3389/fphys.2022.938555 (PMC9326099; doi:10.3389/fphys.2022.938555)
Supplement: Supplementary file 1 [file DataSheet1.PDF]

Supplementary Material

1 Supplementary Figures and Tables

1.1 Supplementary Tables

Supplementary Table 1

Primers used in this study

**Primers for Real-time PCR**	
PxylOR11F	TGGTGGTGTTCGTGGGCTCC
PxylOR11R	CACCCGCCAGACGCTTGTAGAT
PxylActinF	GCCGTCTTCCCGTCCAT
PxylActinR	GATACCTCTCTTGCTCTGGGC
**Specific primers for cloning**	
PxylOR11F	G*ACTAGT*GCCACCATGCAACTACTGAGTAGCATTTGGA (*Spe* I)
PxylOR11R	ATTT*GCGGCCGC*CCTAATCGTCGCTAACTCTGAGCAG (*Not* I)

Note. The restriction enzyme site added to each primer is indicated in parenthesis after the sequence, and the cutting sites are in italics.

Supplementary Table 2

All plant volatile compounds used for the functional characterization of PxylOR11

No.	Name	CAS no.	No.	Name	CAS no.
1	2-Phenylethanol	60-12-8	29	Benzaldehyde	100-52-7
2	β-Citronellol	106-22-9	30	Heptanal	111-71-7
3	Geraniol	106-24-1	31	trans-2-Hexenyl acetate	2497-18-9
4	1-Heptanol	111-70-6	32	cis-3-Hexenyl acetate	3681-71-8
5	(1S)-(-)-Verbenone	1196-01-6	33	1, 4-Diethylbenzene	105-05-5
6	3, 7-Dimethyl-3-octanol	78-69-3	34	4’-Ethylacetophenone	937-30-4
7	(-)-Borneol	464-45-9	35	Tetradecane	629-59-4
8	(+)-Borneol	464-43-7	36	Phenylacetaldehyde	122-78-1
9	(-)-trans-Pinocarveol	547-61-5	37	Nonyl acetate	143-13-5
10	(-)-Linalool	126-91-0	38	Hexyl acetate	142-92-7
11	Linalool	78-70-6	39	Ocimene	13877-91-3
12	Methyl benzoate	93-58-3	40	Tridecane	629-50-5
13	Myrcene	123-35-3	41	Methyl salicylate	119-36-8
14	(R)-(+)-Limonene	5989-27-5	42	(±)-Camphor	76-22-2
15	α-Pinene	80-56-8	43	Nerolidol	7212-44-4
16	(-)-β-Pinene	18172-67-3	44	2, 6-Di-tert-butylphenol	128-39-2
17	Camphene	79-92-5	45	1-Aminoanthracene	610-49-1
18	α-Humulene	6753-98-6	46	2-Pentadecanone	2345-28-0
19	(S)-(-)-Limonene	5989-54-8	47	Cumene	98-86-2
20	α-Terpinene	99-86-5	48	(+)-Cedrol	77-53-2
21	Benzyl alcohol	100-51-6	49	Octyl acetate	112-14-1
22	(-)-trans-Caryophyllene	87-44-5	50	Benzaldehyde	100-52-7
23	(-)-Caryophyllene oxide	1139-30-6	51	Salicylaldehyde	90-02-8
24	Farnesene	502-61-4	52	Ethyl butyrate	105-54-4
25	trans-2-Hexen-1-al	6728-26-3	53	Ethyl hexanoate	123-66-0
26	4-Ethylbenzaldehyde	4748-78-1	54	β-Ionone	14901-07-6
27	3-Vinylbenzaldehyde	19955-99-8	55	(S)-cis-Verbenol	18881-04-4
28	(1R)-(-)-Myrtenal	18486-69-6			
